# Supplementary material for: The nanophthalmos protein TMEM98 inhibits MYRF self-cleavage and is required for eye size specification
Source: PLoS Genet. 2020 Apr 1;16(4):e1008583. doi: 10.1371/journal.pgen.1008583 (PMC7153906; doi:10.1371/journal.pgen.1008583)
Supplement: S1 Table — (DOCX) [file pgen.1008583.s001.docx]

**S1 Table.** **Genotyping primers**

| Primer Name | Sequence (5’-3’) | Product size (allele) |
| --- | --- | --- |
| 1532 | CCAAAGGGGTGCATTTGAAG | 465 bp (WT) |
| 1533 | TGCAAACCCAAGTCAAAAAGC | 595 bp (*tm1c*) |
| 1532 | CCAAAGGGGTGCATTTGAAG | 196 bp (*tm1a*, *tm1b*, *tm1c*, *tm1d*) |
| 1490 | TCGTGGTATCGTTATGCGCC |  |
| 1604 | CCCCCTGAACCTGAAACATA | 310 bp (*tm1b*) |
| 838 | CTCAGACACCCAGCCTTCTC |  |
| 1605 | ACCCTTCTCTCCCTAAGTAGTCT | 867 bp (WT) |
| 1606 | CCCCAAGCCGTCCTTTCC | 1030 bp (*tm1c*)  238 bp (*tm1d*) |
| FLPeF | AGGGTGAAAGCATCTGGGAGA | ~400 bp (FLPe) |
| FLPeR | TCAACTCCGTTAGGCCCTTCA |  |
| 747 | CCTGGAAAATGCTTCTGTCCG | 4 primer reaction |
| 748 | CAGGGTGTTATAAGCAATCCC | 290 bp (control product) |
| 749 | AACACACACTGGAGGACTGGCTA | 450 bp (Cre) |
| 750 | CAATGGTAGGCTCACTCTGGGAG |  |
