# Supplementary figures and images for: The nanophthalmos protein TMEM98 inhibits MYRF self-cleavage and is required for eye size specification
Source: PLoS Genet. 2020 Apr 1;16(4):e1008583. doi: 10.1371/journal.pgen.1008583 (PMC7153906; doi:10.1371/journal.pgen.1008583)

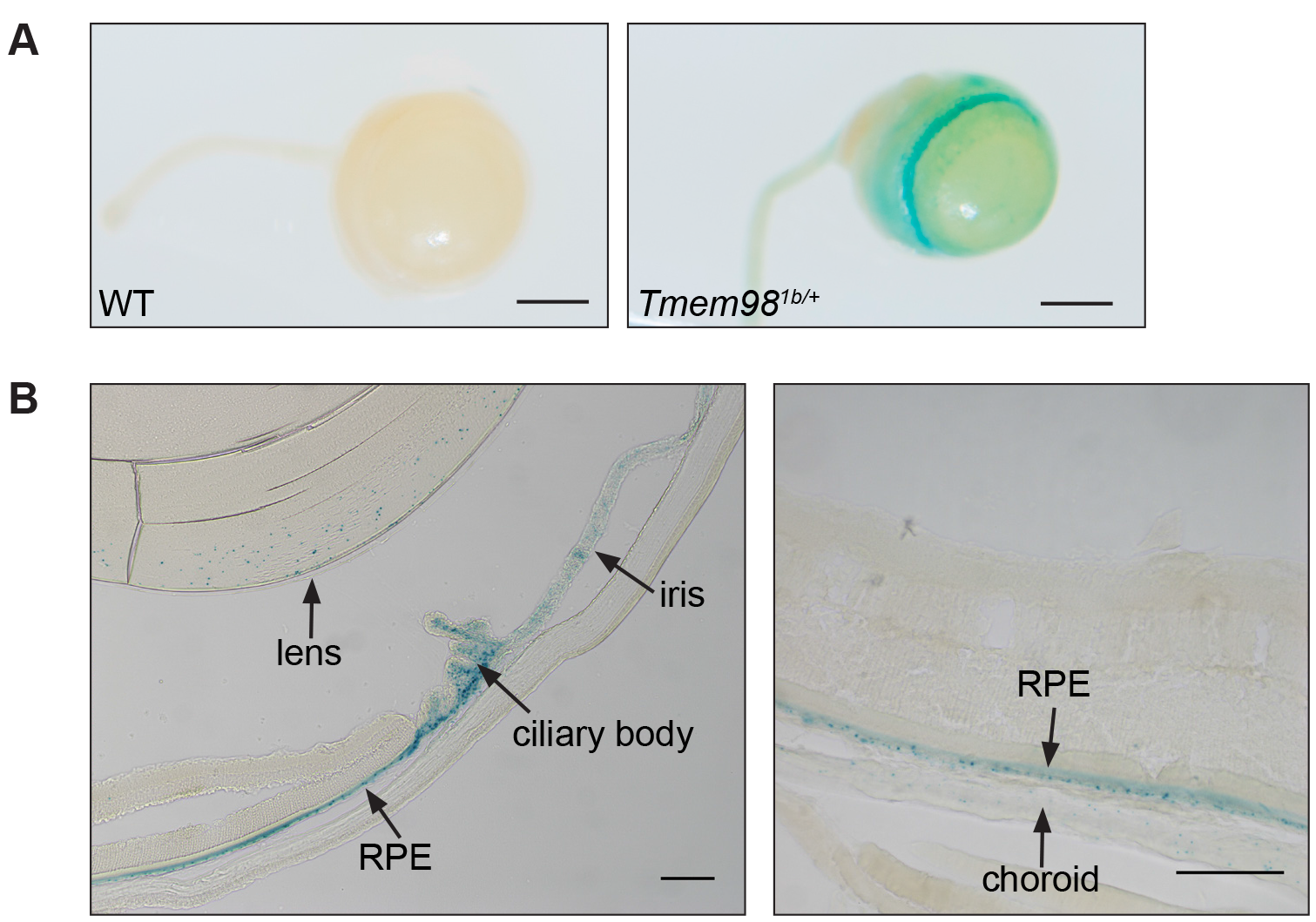

Supplement: S1 Fig — (A) LacZ staining of wild-type (male) and Tmem98tm1b/+ (female) adult albino eyes collected at 5 weeks. The expression pattern of Tmem98 is indicated by the blue staining for the reporter knockout allele Tmem98tm1b. Eyes were enucleated and fixed in 4% PFA/PBS for an hour, rinsed in PBS and washed three times in detergent buffer (0.1 M phosphate buffer pH7.3, 2 mM MgCl2, 0.1% sodium deoxycholate and 0.02% NP-40 (IGEPAL CA-630)). The eyes were then stained in detergent buffer containing 14.5 mM NaCl, 5 mM K3Fe(CN)6, 5 mM K4[Fe(CN)6].3H20 and 150 μg X-gal (5-bromo-4-chloro-3-indolyl-β-D-galactopyranoside) at 37°C protected from light, washed twice in detergent buffer, post-fixed overnight in 4% PFA/PBS, rinsed in PBS and photographed. (B) Cryosections of LacZ-stained Tmem98tm1b/+ (male) adult albino eye collected at 6 weeks show that Tmem98 is strongly expressed in the RPE, ciliary body and iris. There is also some punctate staining in the lens and choroid. Cryosections were prepared as described in Materials and Methods and coverslips mounted in Vectashield (Vector Laboratories) prior to brightfield imaging. Scale bars represent 1 mm (A) and 100 μm (B). (TIF) [file pgen.1008583.s004.tif]

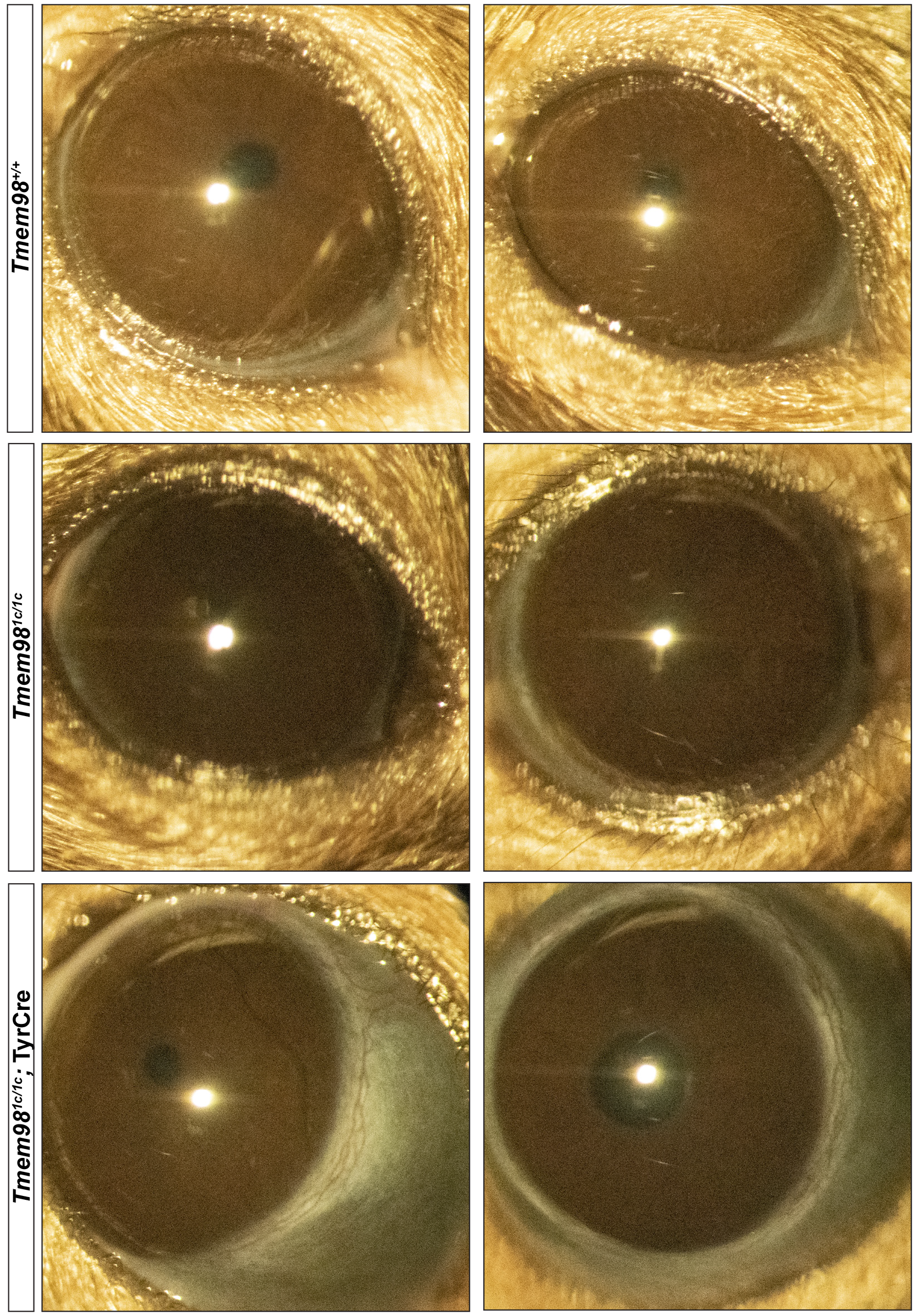

Supplement: S2 Fig — Shown are slit-lamp pictures of eyes from two wild-type mice (top row), two Tmem98tm1c/tm1c mice (middle row) and two Tmem98tm1c/tm1c; Tyr-Cre mice (bottom row). The mice in the top row are a female on the left and a male on the right at 11 weeks of age. The mice in the middle and bottom rows are female 9 week old littermates. Mice homozygous for the floxed conditional allele Tmem98tm1c have eyes of normal size, whereas the eyes of the Tmem98tm1c/tm1c; Tyr-Cre mice are enlarged. (TIF) [file pgen.1008583.s005.tif]

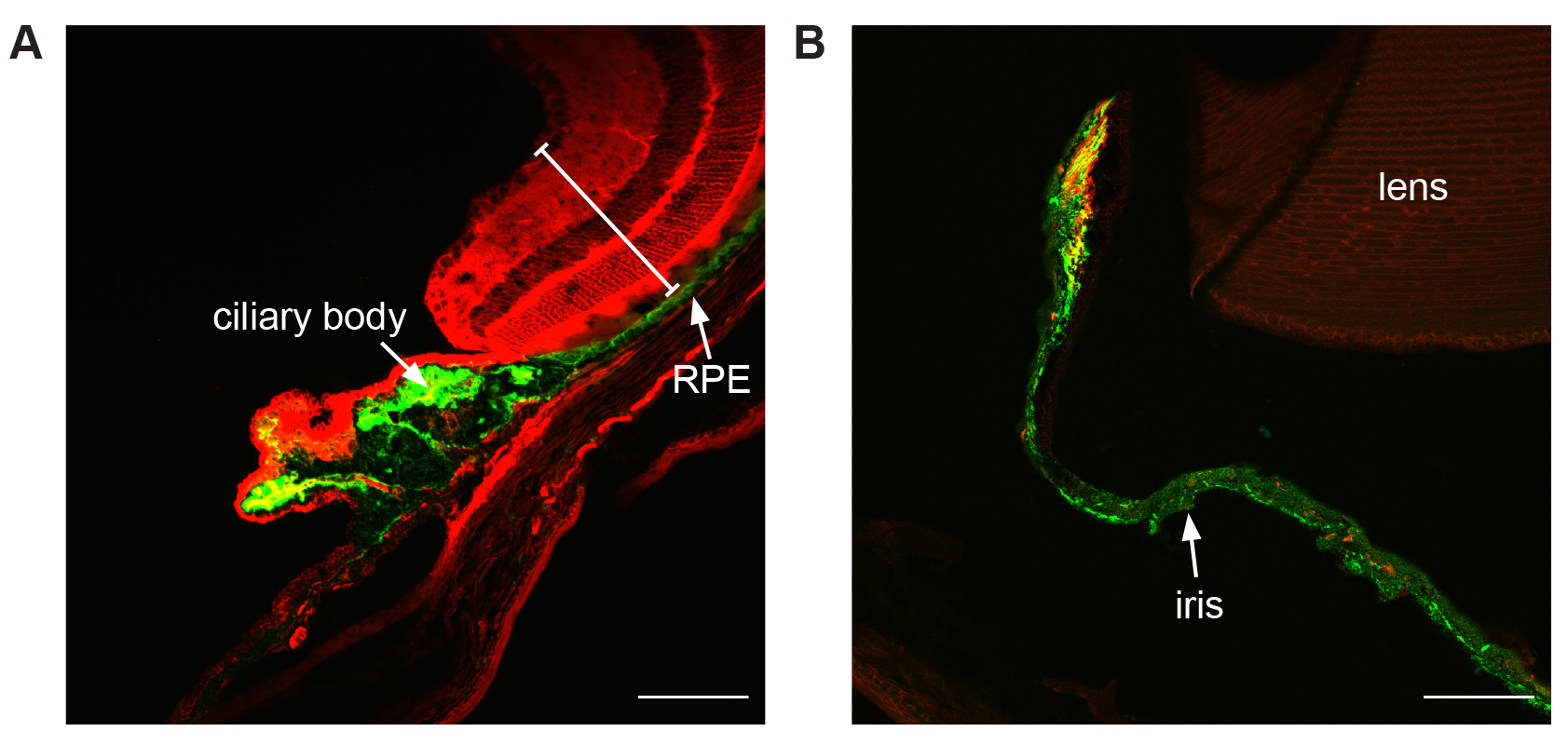

Supplement: S3 Fig — Cryosections of adult R26MTMG; Tyr-Cre eye showing that Cre is expressed in the RPE, ciliary body and iris and not in the neural retina (denoted by a white bar), lens or elsewhere in the eye. Tomato fluorescent protein (red) and green fluorescent protein (green). Scale bars represent 100 μm. (TIF) [file pgen.1008583.s006.tif]

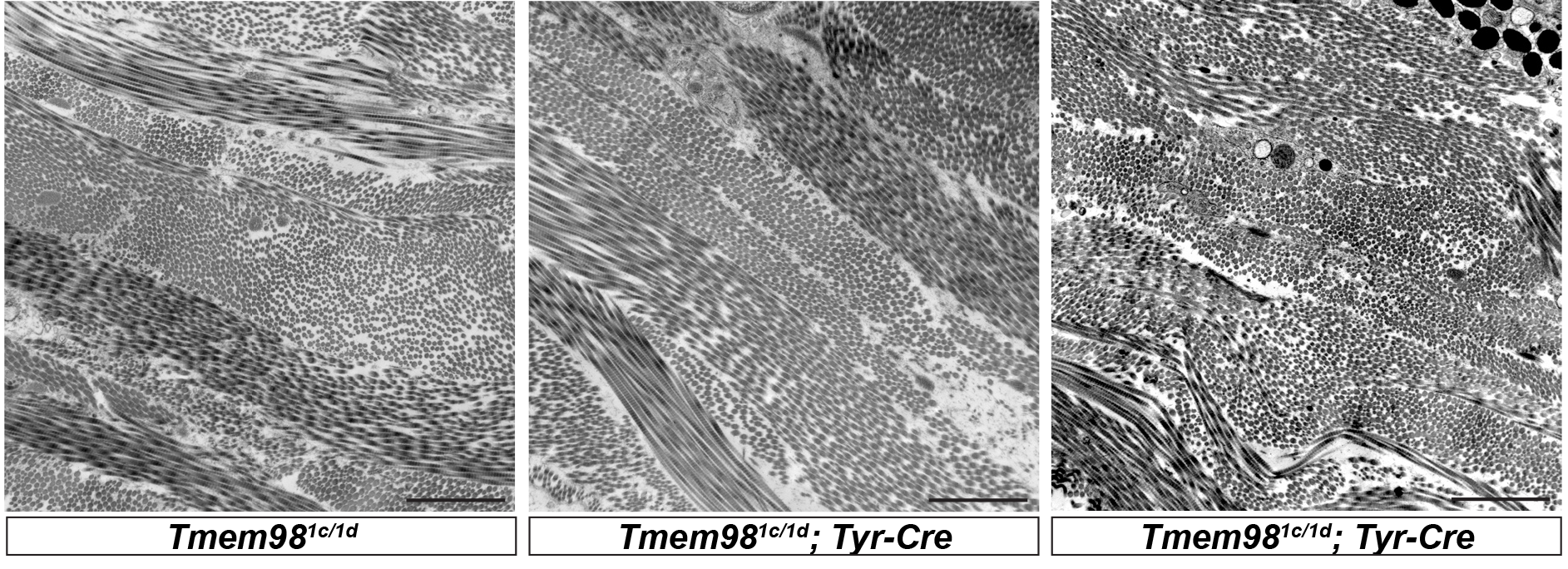

Supplement: S4 Fig — Eyes from four month old male littermate mice were fixed overnight in 3% glutaraldehyde in cacodylate buffer at 4°C then post-fixed in 1% osmium tetroxide for two hours at 4°C. After dehydration through ascending grades of alcohol and propylene oxide they were impregnated with TAAB Embedding Resin (medium grade premix) and cured for 24 hours. Ultrathin sections were stained with uranyl acetate and lead citrate and viewed on a JEOL JEM 1200 EX2 transmission electron microscope fitted with an AMT Digital Camera using the AMTv600 image capture software. There does not appear to be any difference in the collagen bundle structure between the control Tmem98tm1c/1d (left) and mutant Tmem98tm1c/tm1d; Tyr-Cre (centre and right). Scale bars represent 2 μm. (TIF) [file pgen.1008583.s007.tif]

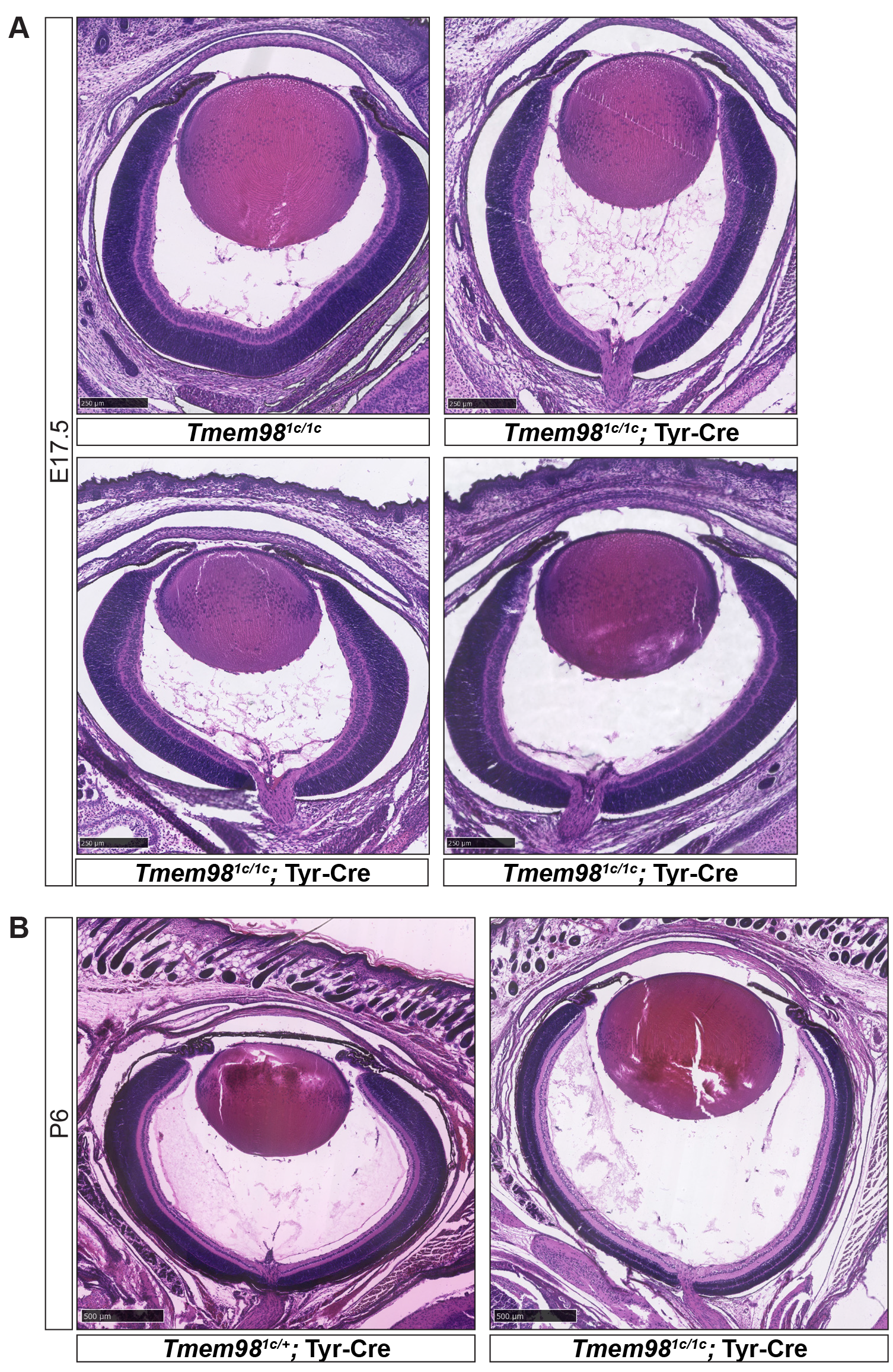

Supplement: S5 Fig — H&E stained head sections are shown. (A) Sections through the eye for one control Tmem98tm1c/tm1c and three mutant Tmem98tm1c/tm1c; Tyr-Cre E17.5 embryos. The eye shape is noticeably elongated in one of the Tmem98tm1c/tm1c; Tyr-Cre embryos (top right) compared to the control (top left). (B) Sections through the eye for control Tmem98tm1c/+; Tyr-Cre (left) and mutant Tmem98tm1c/tm1c; Tyr-Cre P6 mice. The posterior segment of the mutant Tmem98tm1c/tm1c; Tyr-Cre eye is expanded and the retinal layers are thinner compared to the control. For P6 samples mice were culled and following decapitation and removal of the lower jaw heads were fixed in Davidson’s fixative at 4°C. Otherwise they were processed as described for embryos in Materials and Methods except that they were sectioned at 16 μm. Scale bars represent 250 μm (A) and 500 μm (B). (TIF) [file pgen.1008583.s008.tif]

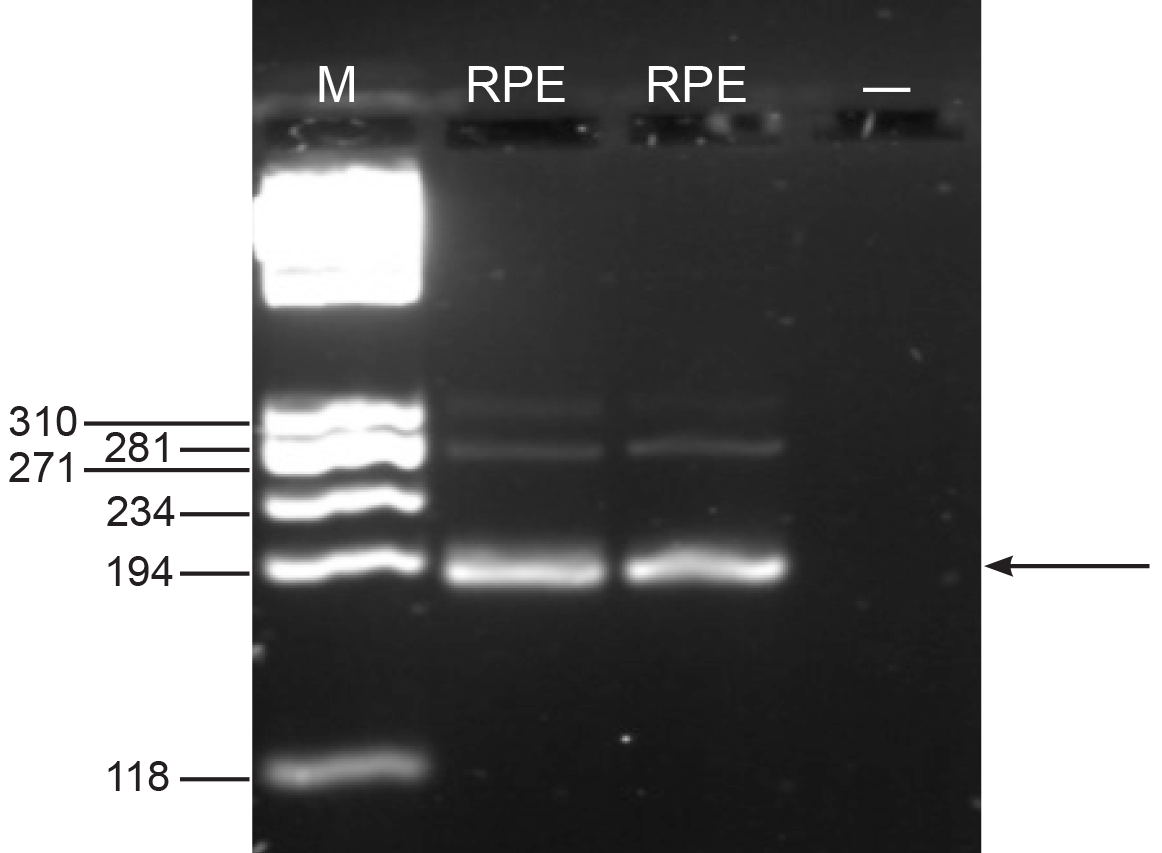

Supplement: S6 Fig — Shown is RT-PCR analysis of RPE collected from two C57BL/6 mice. The forward primer used was from exon 17 (5’-TAGCTCTGGTGGTGGTCATG-3’) and the reverse primer spanned the exon 20/21 boundary (5’-GTAACCAGCAGCAAAGAGGG-3’). The predicted RT-PCR product sizes are 268 bp if exon 19 is included and 187 bp if exon 19 is excluded. The predominant splice form present in RPE is 187 bp (arrowed) which lacks exon 19. This was confirmed by sequencing. The sizes of the DNA fragments in the marker lane (M) are indicated to the left. The lane labelled with dash is a no template control. RNA was prepared using an RNeasy Plus Micro kit (Qiagen) following the manufacturer’s instructions and first strand cDNA was prepared using a GoScript Reverse Transcription System (Promega). (TIF) [file pgen.1008583.s009.tif]

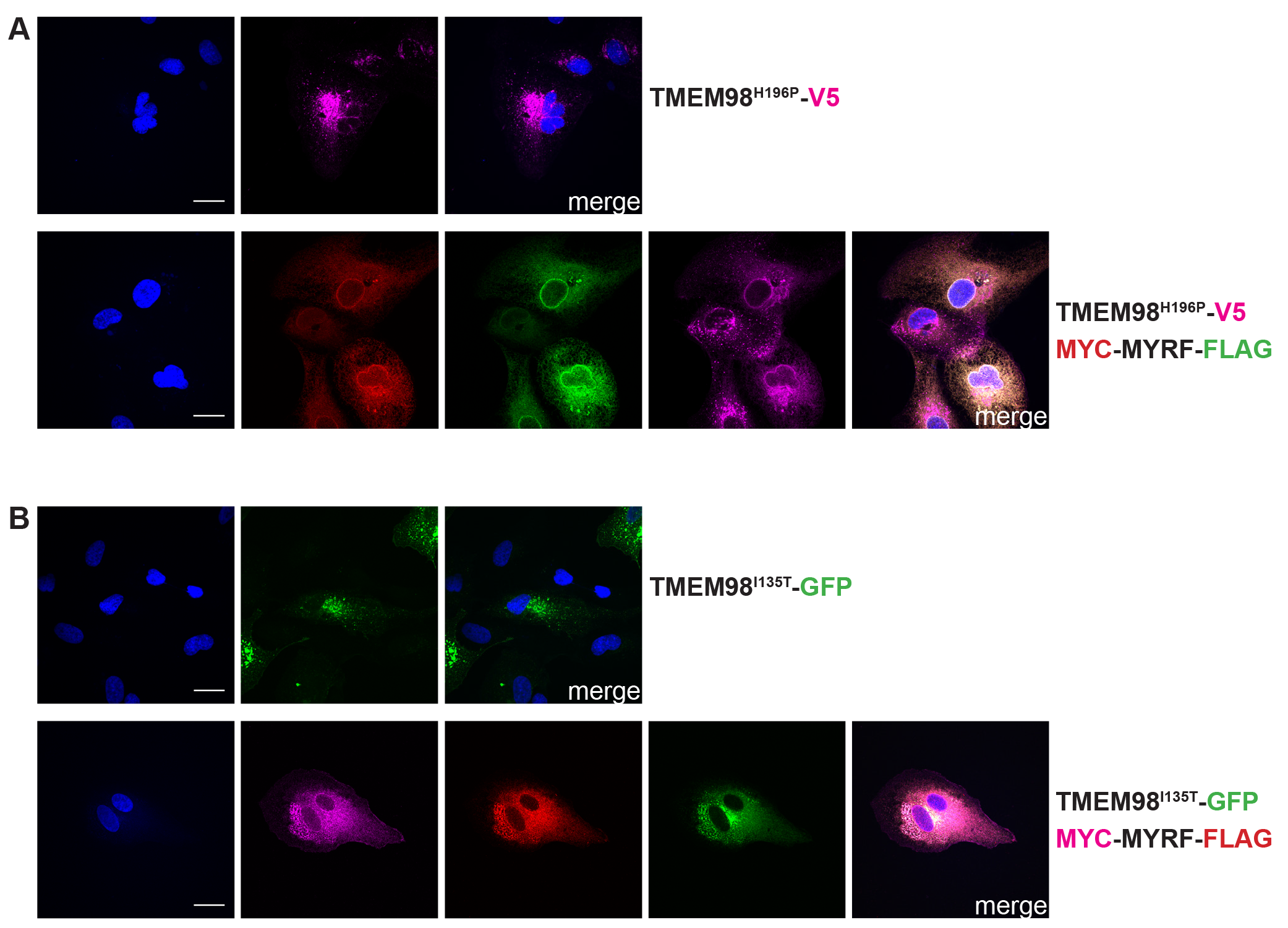

Supplement: S7 Fig — (A) ARPE-19 cells were transiently transfected with TMEM98H196P-V5 alone (top) or with MYC-MYRF-FLAG (bottom) and immunostained with anti-V5 (magenta), anti-MYC (Cell Signaling Technology, 2278) (red) and anti-FLAG (Biolegend, 637302) (green) antibodies as indicated. DAPI staining is in blue. When MYC-MYRF-FLAG is co-transfected with TMEM98H196P-V5 it remains intact and colocalises with TMEM98-V5 in the membrane. The TMEM98H196P-V5 construct was made in the same way as the TMEM98-V5 construct except that Tmem98 open reading frame with the initiating ATG was amplified from cDNA isolated from Tmem98H196P/H196P mice. (B) ARPE-19 cells were transiently transfected with TMEM98I135T-GFP alone (top) or with MYC-MYRF-FLAG (bottom) and immunostained with anti-MYC (Cell Signaling Technology, 2276) (magenta) and anti-FLAG (Cell Signaling Technology, 2368) (red) antibodies as indicated. TMEM98I135T-GFP is in green and DAPI staining is in blue. When MYC-MYRF-FLAG is co-transfected with TMEM98I135T-GFP it remains intact and colocalises with TMEM98I135T-GFP in the membrane. To make the TMEM98I135T-GFP construct the Tmem98 open reading frame with the I135T missense mutation was amplified by PCR using the primers 5’- GGGAGATCTCCCGGCATGCCCTGCTGCTGG-3’ and 5’- CCCACCGGTATGGCCGACTGTTCCTGCAG -3’ and cloned into the BglII and AgeI sites of pEGFP-N1 (BD Biosciences Clontech). Scale bars represent 20 μm. (TIF) [file pgen.1008583.s010.tif]

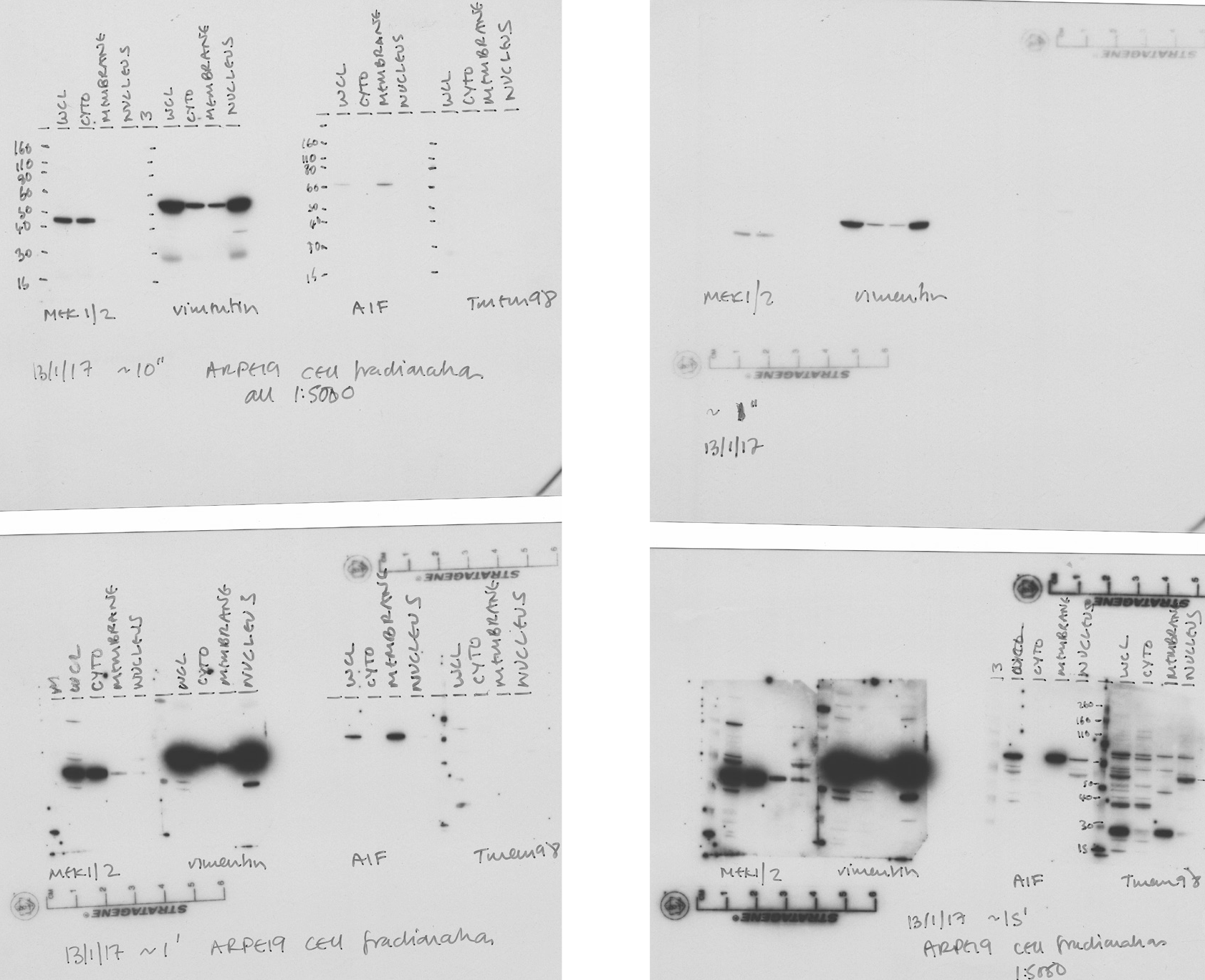

Supplement: S8 Fig — Uncropped images of the Western blots used to make Fig 6A. (TIF) [file pgen.1008583.s011.tif]

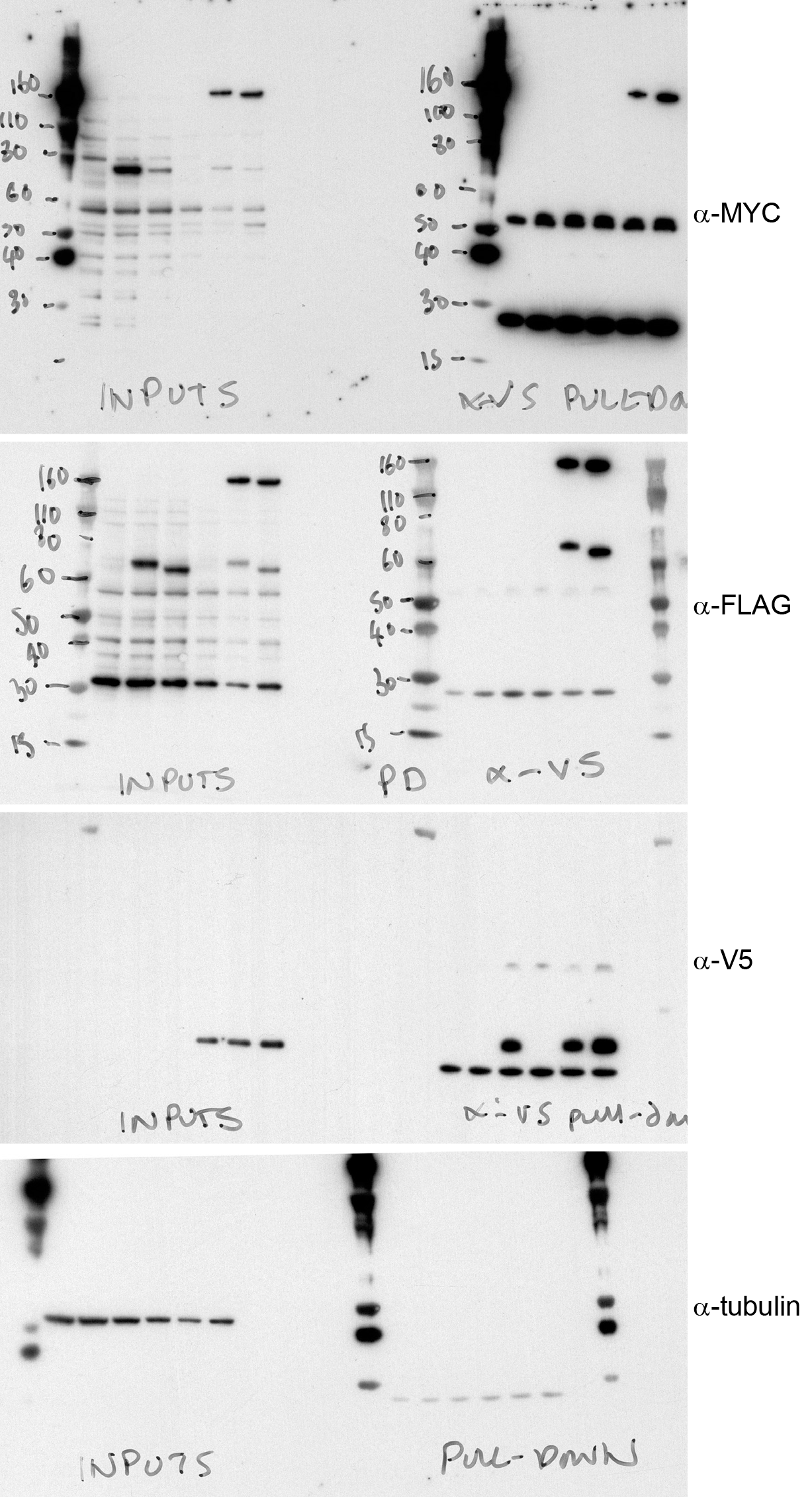

Supplement: S9 Fig — Uncropped images of the Western blots used to make Fig 7A. The antibodies used are indicated to the right of the images. (TIF) [file pgen.1008583.s012.tif]
