# Supplementary material for: The nanophthalmos protein TMEM98 inhibits MYRF self-cleavage and is required for eye size specification
Source: PLoS Genet. 2020 Apr 1;16(4):e1008583. doi: 10.1371/journal.pgen.1008583 (PMC7153906; doi:10.1371/journal.pgen.1008583)
Supplement: Attachment — Submitted filename: response_to_reviewers.doc [file pgen.1008583.s013.doc]

Greg Barsh,

Editor-in-Chief,

PLoS Genetics

17^th^ February 2020

Dear Greg,

Thank you for sending the reviews of our paper “The nanophthalmos protein TMEM98 inhibits MYRF self-cleavage and is required for eye size specification”. I am very pleased that the referees found the manuscript interesting. They both made a number of relevant and valid points and we have amended the paper in line with their recommendations. The changes are outlined below and we have endeavoured to answer their comments and queries which I have included (blue text).

All numerical data underlying the graphs and summary statistics are now included as Supporting Information (S1 Dataset (lines 182,190 and 516) and S2 Dataset (line 987)). The mass spectrometry proteomics data have been deposited to the ProteomeXchange Consortium and will be publicly available once the paper has been accepted for publication (lines 678-680). All the figures were uploaded to the PACE digital diagnostic tool.

I would like to thank the reviewers for their comments and I very much hope that you find the revised paper acceptable for publication in PLoS Genetics.

I look forward to hearing from you,

Best wishes

Sally

Dr Sally H. Cross

Senior Investigator Scientist

sally.cross@igmm.ed.ac.uk

Reviewer #1: Cross et al. presented the ocular phenotypes of a mouse when Tmem98 was knocked out in RPE and provided further evidence of the interaction of TMEM98 and MYRF in this well written manuscript. The authors presented strong rationale to investigate the function of TMEM98 given its association with eye size in human patients. On the first glance, it is confusing as the mutations in TMEM98 cause nanophthalmos is human patients, the overwhelming phenotype in mouse with RPE specific Tmem98 KO is axial enlargement of the eye. However, it is not uncommon at all, even just in human patients. Some mutations in the same gene can cause opposite phenotypes. This perhaps should be added to the discussion. In addition, mouse models often fail to recapitulate human diseases.

We agree with the reviewer both that mutations in the same gene can cause opposite phenotypes and that often mouse models do not recapitulate human diseases. We have mentioned both these points in the paper. In the Discussion we refer to work on *PRSS56*. Mutations in this gene can cause recessive nanophthalmos and variants of this gene are associated with myopia (page 16, lines 363-367). In the Introduction we refer to work were mouse models have been made in genes associated with nanophthalmia in humans which found that, rather than affecting eye size, the predominant result was retinal abnormalities (pages 4-5, lines 90-94). In the specific case of *TMEM98* we would argue that the human missense mutations are not loss-of-function, as outlined in our previous paper (Cross *et al.*, Missense Mutations in the Human Nanophthalmos Gene *TMEM98* Cause Retinal Defects in the Mouse IOVS 60, 2875-2887 (2019)).

The evidence of interaction between TMEM98 and MYRF is convincing, yet not terribly exciting given the recent publication in this specific journal by Garnai et al. (PLoS Genet. 2019 May; 15(5): e1008130). The idea of interaction of RPE with sclera in the setting of eye development and size is very intriguing and brought out and studied by the authors, but it falls short (see comments below), which is somewhat disappointing.

Line 100-102: "thus identifying a pathway..." seams a bit strong as this has been previously shown by Garnai et al. Adding further evidence should be considered.

We agree that “identifying” is a bit strong given the recent published work. We have amended the sentence to say that our studies confirm the pathway involving *TMEM98* and *MYRF* in eye size specification (page 5, lines 103-104).

Line 114: "the eyes of majority (5/6) KO mice" is not clear. When the mouse had elongated eye, was it for both eyes? 5/6 refers to 5 out of 6 eyes or mice? I suppose it means 5/6 mice. If so, was the elongation similar in both eyes from the same mouse?

We apologise for the confusion. 5/6 refers to the number of mice. We have amended the sentence to make this clear (page 6, lines 116-117). It was not always possible to ascertain the elongation in both eyes depending on the angle of cutting but when we could determine the eye shape in both eyes of a mouse they were similar.

line 149-153: given the lack of quantification and relatively small number of animals, it is hard to be totally convinced on the reduction of autofluorescence. Similar to the comments above, were two eyes from the same mouse similar?

Yes, both eyes from the same mouse were similar.

Line 173-175: the sclera appears very thin in mutant mice. Although the authors stated that they did not observe any obvious abnormalities by TEM, was there some subtle disorganized structure as shown in S4? Was TEM looking at collegian fibrils good enough or other studies should be conducted? It is a missed opportunity to not study sclera in details. Rather, the authors went on to show ERG changes. Such change should not be a surprise. This does not add much significance of this study, hence it should be moved to supplemental materials.

We agree that the sclera was very thin in the mutant eyes and now emphasise this point (page 2 line 28, page 8 lines 175-178 and page 15 lines 335 and 350-351). By TEM we did not observe any striking abnormalities in the collagen fibrils (S4 Fig). Investigation of the sclera will be the subject of future work (page 15, lines 353-354).

Line 336-338: "numerous studies", references should be given. This is very intriguing and should be further studied.

References 44-47 have been added (page 15 line 342).

Lone 384-386: It indeed would provide ultimate evidence if Myrf KO could rescue the enlarged eye phenotype of Tmem98 KO. Do the authors have the plan to make the double KO mouse? Any data? As the phenotype can be characterized in embryos, it may not be a very difficult and time consuming experiment.

We would very much like to do this experiment. Creating/importing the *Myrf* conditional KO and crossing with our *Tmem98* conditional KO will take some time and is part of our future plans but beyond the scope of this paper.

Line 411: R26MTMG mice: delete mice.

This has been done (page 18 line 417).

Figure 2: Did authors measure white to white distance? What about corneal thickness by OCT? It would add more evidence that anterior segments are normal.

We did not measure white to white distance. We have measured the corneal thickness in histological sections and OCT and found no significant difference in corneal thickness between mutant and control. This is now included in the paper (page 9 lines 189-191).

Fig 3. The emphasis is on the retina thinning. However, the other structures, such as choroid and sclera are vastly abnormal too, which could play a important role in globe elongation.

We now highlight in the figure legend for Fig 3 the abnormalities in the choroid and sclera (page 38 lines 979-980).

Fig 4. should be moved to supplemental materials.

We would like to keep this figure in the main paper rather than have it as a supplemental figure as it demonstrates that the mice have essentially no visual function.

Fig. 6B. Information appears scant. N of ? Difference between top and bottom panels?

Fig 6B shows the results of a cell transfection experiment. Shown are representative transfected cells as indicated by GFP fluorescence (green) under non-permeabilised (left) or permeabilised (right) conditions. All transfected cells observed looked similar. The cells on the top row have been stained with an anti-V5 antibody (red) and the cells on the bottom row have been stained with an anti-GFP antibody (red). The anti-GFP antibody can only access its epitope when the cells have been permeabilised indicating that its epitope is located on the inside of the cell. The anti-V5 antibody can access its epitope whether or not the cell has been permeabilised indicating that its epitope is extracellular. We have amended the figure legend to clarify what was done (page 40 lines 1022-1030).

S1 Fig. it appears Tmem98 is also expressed in lens and choroid???

The punctate staining in the lens and choroid is now mentioned in the figure legend and they are labelled in the figure (page 43 lines 1088-1089). We would emphasise that the RPE, ciliary body and iris are the strongest sites of *Tmem98* expression. Note that we did not observe any LacZ staining in the lens at E16.5 (Fig 1).

S3 Fig. legend: RPE should be changed to iris.

This has been done (page 43 line 1101).

S5 Fig. Given the fact the contact lenses have to be changed because of elongation of the eye in mutant mice, this reviewer questions the reliability of comparison between control and mutant. Why there is such large variation in temporal retina in both control and mutant mice? One male and two females at age between 10-14 weeks were used. Sex and age (4 weeks difference) could play a role??? Consider remove this figure as it does not add much more information.

This figure has been removed as suggested.

S6 Fig: lens in the mutation in middle and bottom panel is larger than corresponding control in this figure, which may indicate the pariticular section shown here is more central, wihle the control is not. This could result in larger appearance of the eye, as often seen in histology.

To ensure equivalent positioning for different samples we show sections that go through the optic nerve, if possible. In both panels the sections shown all go through the optic nerve. We do not observe any lens differences of note between mutant and controls.

Reviewer #2: This is a well written study of a new animal model, a conditional knockout of TMEM98 in the retinal pigment epithelium, which gives insight into one possible contributory factor myopia. That is important because myopia is a major clinical burden and is increasing in the human population for reasons only partially understood; and because this finding gives rare functional insight into the mechanism by which SNPs detected in a GWAS contribute to a human phenotype. There were a few sentences I could improve for readability - the authors don't seem to like using commas much - but I cannot suggest improvements that are beyond the brief of a copy editor.

We have broken up some long sentences and added some commas which I hope will improve readability.

In an ideal world it would have been nice to explore why these mutations cause different phenotypes in mice and humans, and why some TMEM98 variants increase eye size and others reduce it. Their use of the word "unexpectedly" in describing what they found serves to underline this complex question. For the latter point they hypothesize in discussion that the human nanophthalmos-causing mutations are gain of function, and say as evidence that a compound het mouse with complete knockout and missense had no phenotype, so each compensates for the other. There is no reference for this missing bit of data - was it in the previous paper? Anyway that does not explain why key missesne variants or deletion of the C terminus cause nanophthalmos in humans, while complete knock-out increases the posterior eye size in mice.

The evidence for the nanophthalmos-associated missense mutations being gain-of-function is in our previous paper (Cross et al., Missense Mutations in the Human Nanophthalmos Gene TMEM98 Cause Retinal Defects in the Mouse IOVS 60, 2875-2887 (2019)).

But I assume these puzzles will be the target of their future work. As a self-contained project the analysis of this mouse conditional knockout is beautifully documented, thoroughly described, and does contain original and important new findings. The abstract is slightly confusing - if I read it correctly the novelty is not in finding that TMEM98 inhibits the self-cleavage of MYRF, documented in ref 49, but that this interaction is so crucial in determining eye size.

We have amended the abstract to emphasise this very point (page 2 lines 36-37).
